# Supplementary figures and images for: Schistosoma mansoni Larval Extracellular Vesicle protein 1 (SmLEV1) is an immunogenic antigen found in EVs released from pre-acetabular glands of invading cercariae
Source: PLoS Negl Trop Dis. 2021 Nov 18;15(11):e0009981. doi: 10.1371/journal.pntd.0009981 (PMC8639091; doi:10.1371/journal.pntd.0009981)

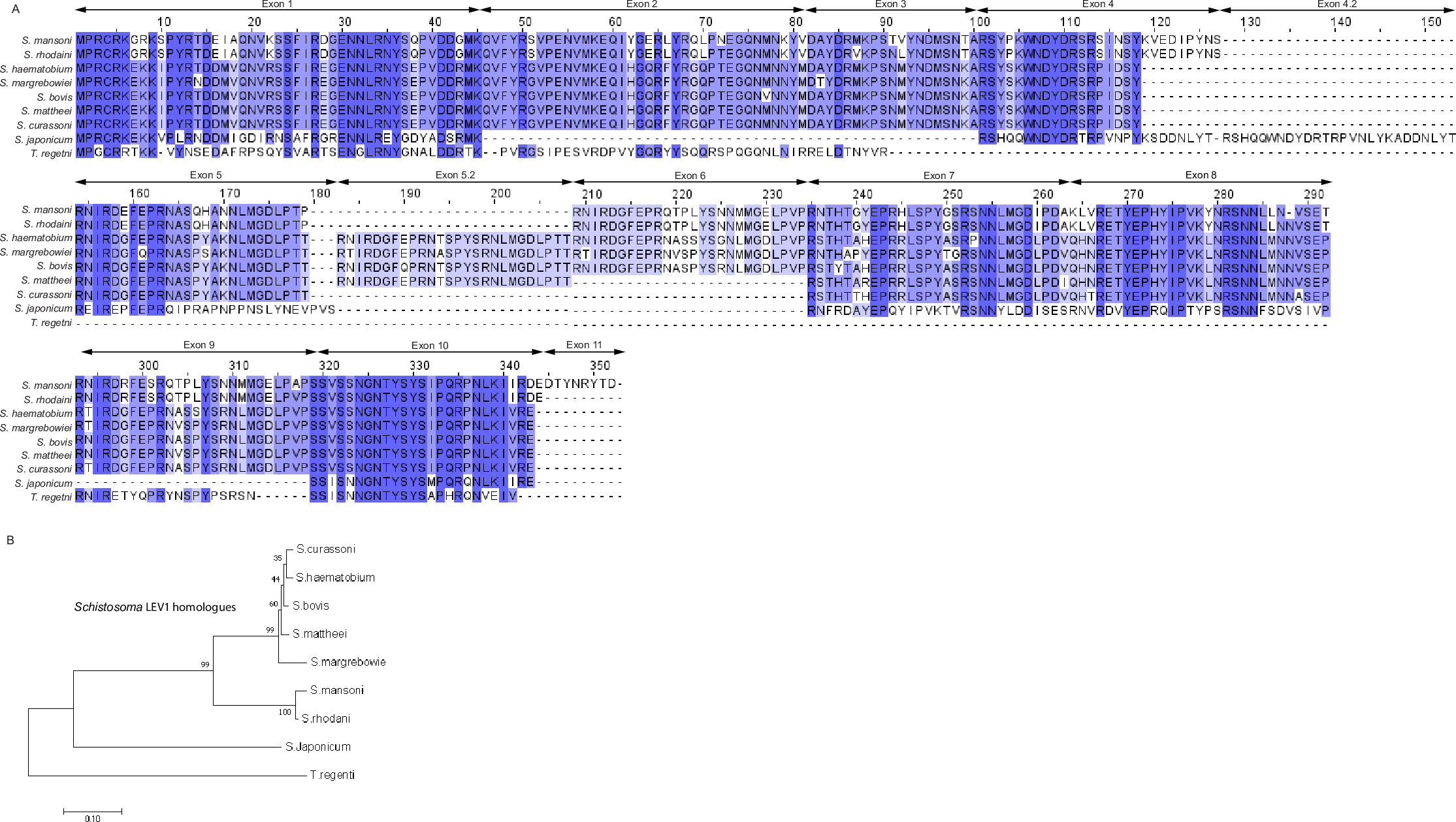

Supplement: S1 Fig — (A) MUSCLE Alignment of the deduced full-length amino acid sequences of LEV1 homologues (S1 Table) visualised by JALview [67]. Conserved amino acid residues are shaded blue, residues with a positive BLOSUM62 score are shaded lilac, non-conserved residues are white. Exon numbers (relative to the full-length SmLEV1.1) are indicated above, and probable exon duplications (identified via tBLASTn) named Exon#.2. (B) Phylogenetic tree of deduced LEV1 homologues was inferred by using the Maximum Likelihood method based on the JTT matrix-based model [68] and drawn using MEGA7 (http://www.megasoftware.net/). The tree with the highest log likelihood (-2154.30) is shown. Node values indicate percentage of trees in which the associated taxa clustered. The tree is drawn to scale, with branch lengths measured in the number of substitutions per site. (TIF) [file pntd.0009981.s001.tif]

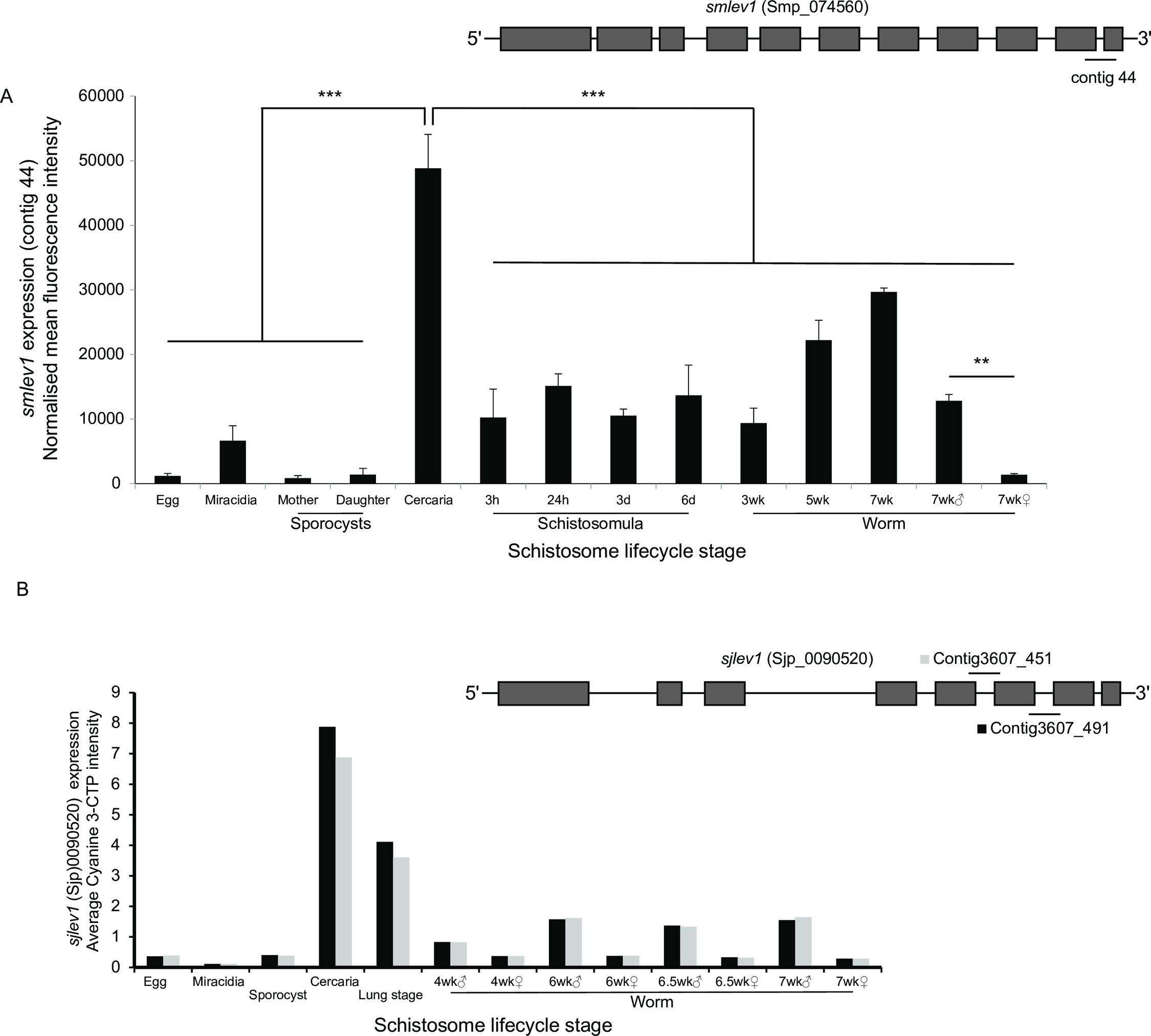

Supplement: S2 Fig — (A) Analysis of data derived from a previous DNA microarray study [19] quantitatively shows a significant abundance of smlev1 in cercaria compared to all other lifecycle stages, and a 10.7-fold higher expression in the male vs female. Contig_44 represents a 50 bp oligonucleotide spanning a region comprised of exon 10 and 11. The specific life-cycle stage is shown along the x-axis, mean normalised fluorescence units is shown along the y axis. Error bars represent standard error of the mean normalised fluorescence units. Significance compared to cercaria stage is indicated as: p<0.001 ***; p<0.005 **. (B) The S. japonicum lev1 homologue (Sjp_0090520) exhibits developmentally regulated expression similar to smlev1. Data derived from a previous S. japonicum microarray study by Gobert et al [20] shows the sjlev1 homologue also exhibits developmentally regulated expression. Contig3607_451 (grey) represents a 60 bp oligonucleotide spanning exon 5 and 6 (equivalent to smlev1 exon 8 and 9), whilst Contig3607_491 (black) represents a 60 bp oligonucleotide spanning exon 6 and 7 (equivalent to smlev1 exon 9 and 10); the sequences detected by these two probes overlap each other by 20 base pairs. The specific life-cycle stage is shown along the x-axis, Average Cyanine 3-CTP intensity is shown along the y axis. (TIF) [file pntd.0009981.s002.tif]

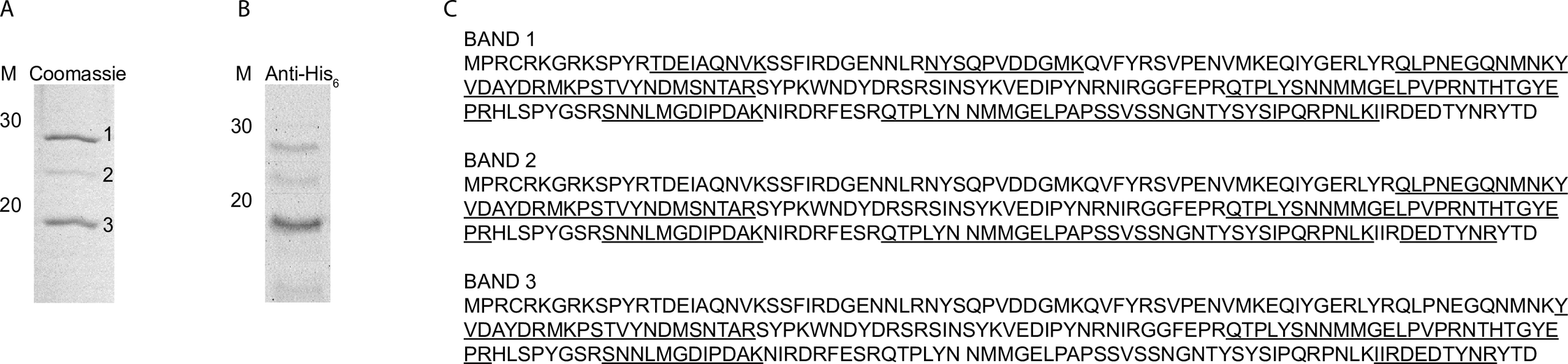

Supplement: S3 Fig — rSmLEV1.3 production resulted in three protein bands <30 kDa in size visualise by SDS-PAGE and either stained by (A) Coomassie blue or transferred to PVDF and probed via (B) anti-His6 antibody (used at 1:20,000). All bands contained peptides identified as SmLEV1.3 (Smp_074560.3) and no other SmLEV1 isoform, or E. coli proteins, when subjected to BLAST analysis against the GenBank database (p<0.05) (available at ncbi.nih.gov) (S2 Table). (C) Mapping peptides to the sequence (underlined) suggests that bands 2 and 3 represent successive N-terminal breakdown of rSmLEV1.3. M—protein standard, mass in kilo Daltons. (TIF) [file pntd.0009981.s003.tif]

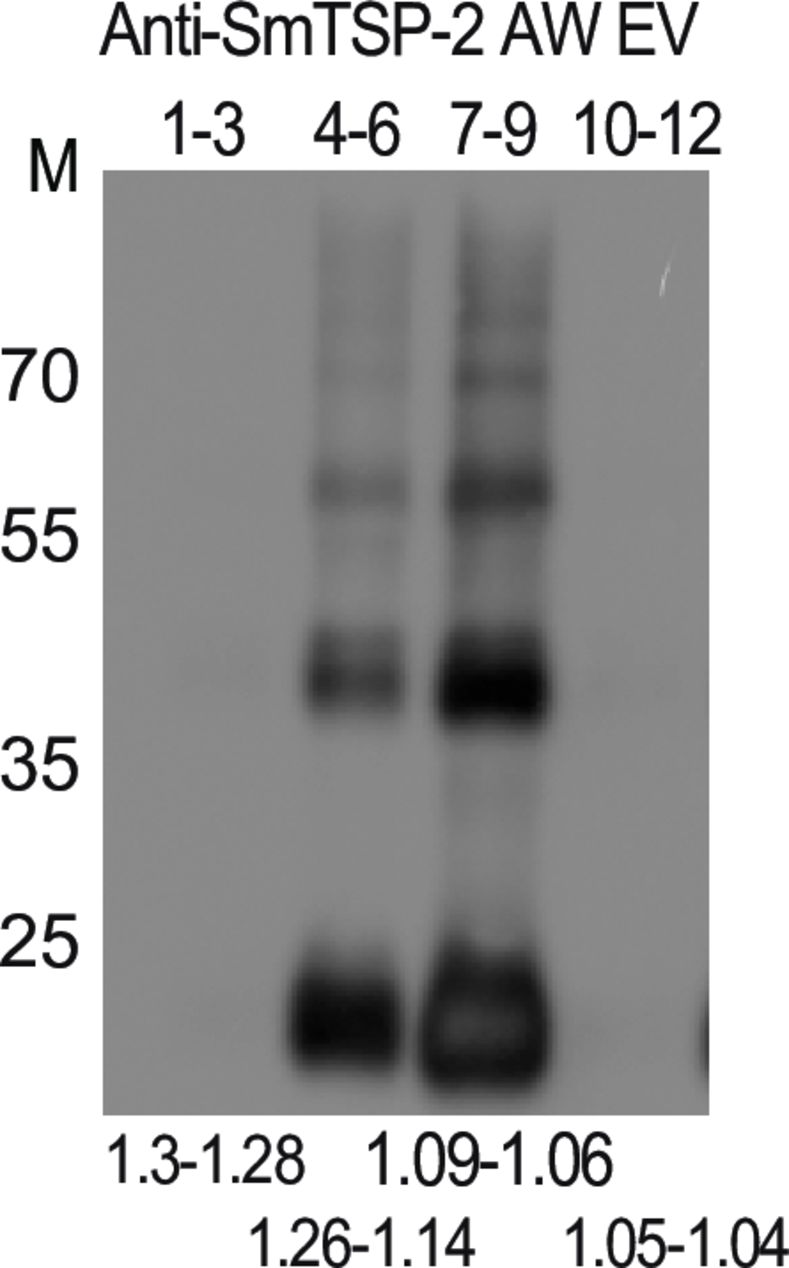

Supplement: S4 Fig — EVs from mixed-sex S. mansoni adult worms were isolated and subjected to density gradient separation as described, before probing with anti-SmTSP-2 Ab (1:2,000). Fractions containing SmTSP-2 (4–9) were combined and used for probing with anti-SmLEV1 pAb. Fraction numbers are indicated above the blot; the respective density ranges in g/cm3 are shown below the plot. Secondary anti-rabbit HRP conjugated antibody was used at 1:10,000. M = protein standard, mass in kilo Daltons. (TIF) [file pntd.0009981.s004.tif]

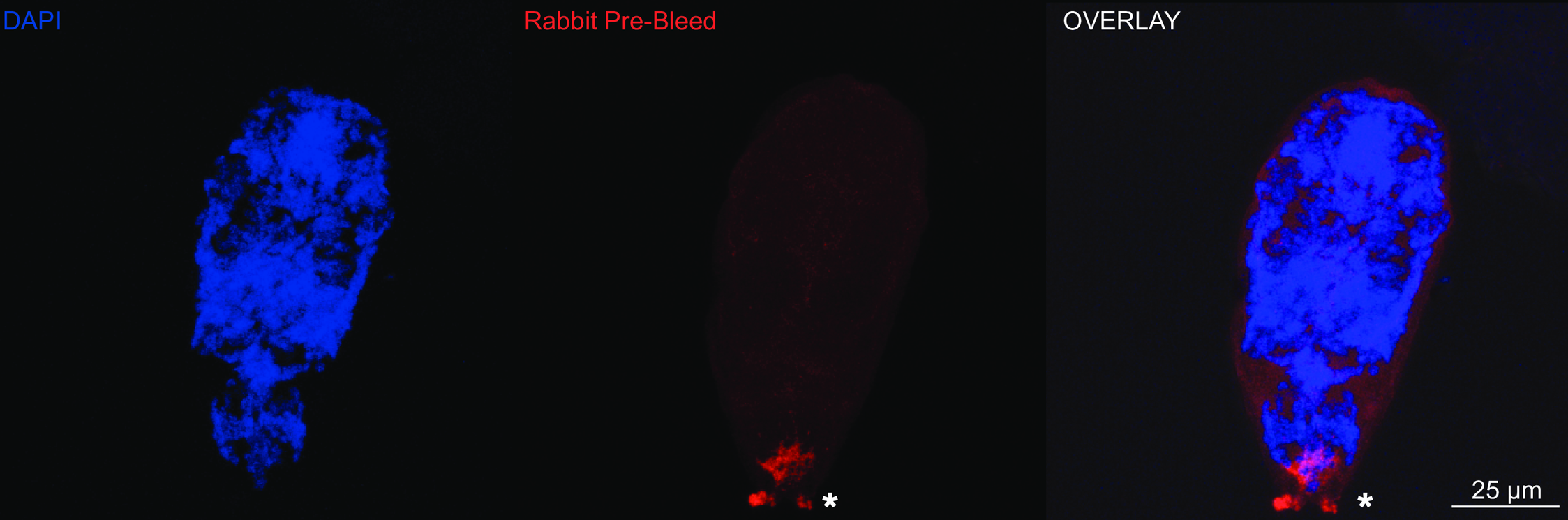

Supplement: S5 Fig — Permeabilised cercariae were stained with rabbit pre-bleed serum (1:50) followed by goat anti-rabbit Alexa Fluor 594 secondary antibody (Fisher Scientific, 1:500), then PBSTx containing DAPI (1 μg/mL). Cercariae were mounted and visualised on a SP8 Leica super resolution confocal microscope. Asterisk indicates staining of peripheral extensions also seen after staining with anti-SmLEV1.3 (Fig 6). (TIF) [file pntd.0009981.s005.tif]

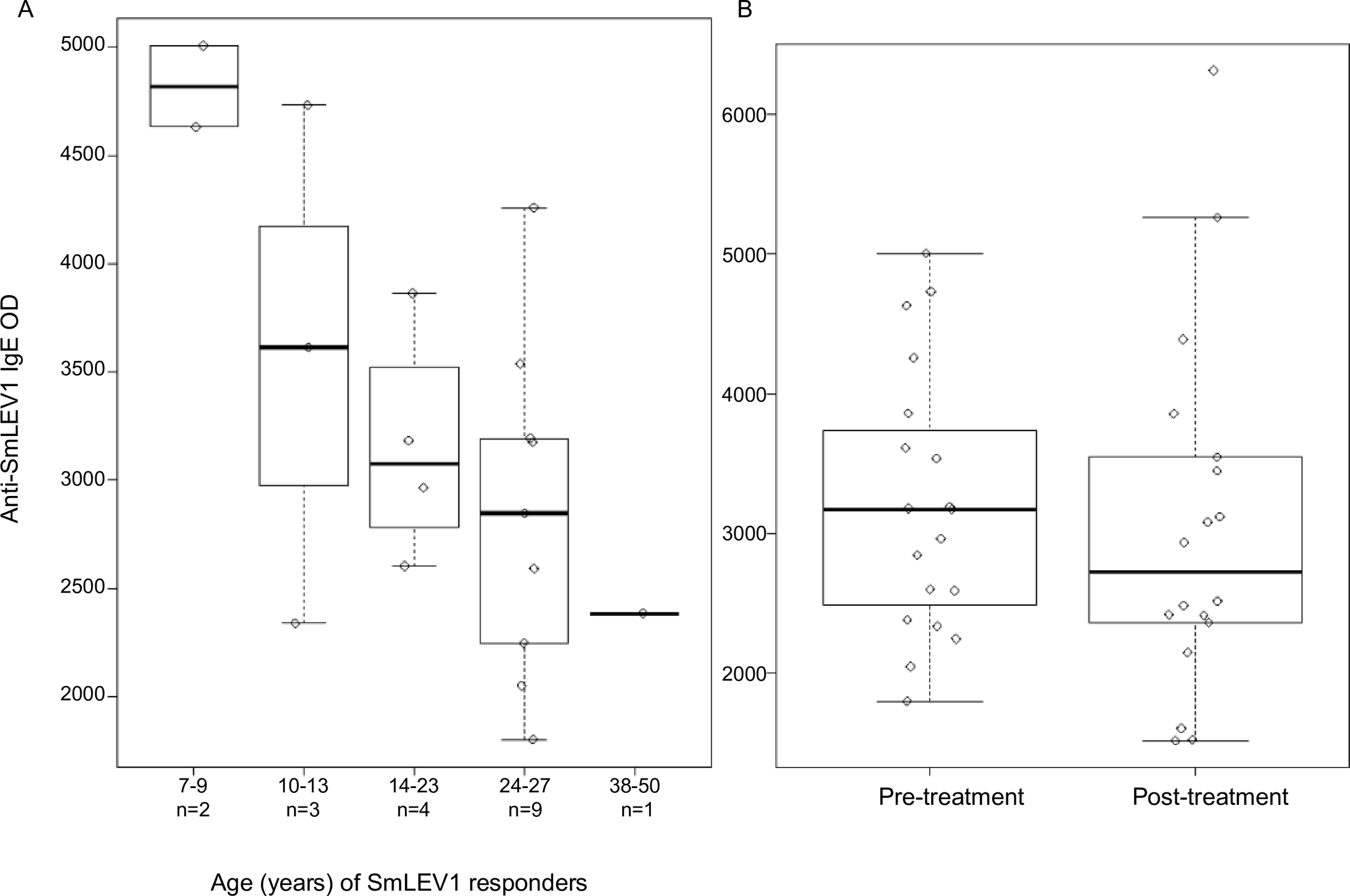

Supplement: S6 Fig — Descriptions of cohort selection, quantitative parasitology, and treatment regimens for this study can be found in a previous publication [25]. rSmLEV1.3 was probed with biotinylated mouse anti-human IgE (Pharmingen). Detection was achieved with HRP-conjugated streptavidin (1:3,000 Mast Group Ltd), followed by development in o-phenylenediamine substrate solution (Sigma). (A) SmLEV1 IgE responders made up 11% of the Ugandan male cohort. The anti-SmLEV1.3 IgE response decreased with age and (B) no significant drop is seen in the anti-SmLEV1.3 IgE response after treatment. The number and percentage of responders are shown for each age group. The boxplot shows the median (line) with the upper and lower quartile (box) as well as the upper and lower observation within 1.5x interquartile range (IQR) (whiskers). Individual data points are also plotted. (TIF) [file pntd.0009981.s006.tif]

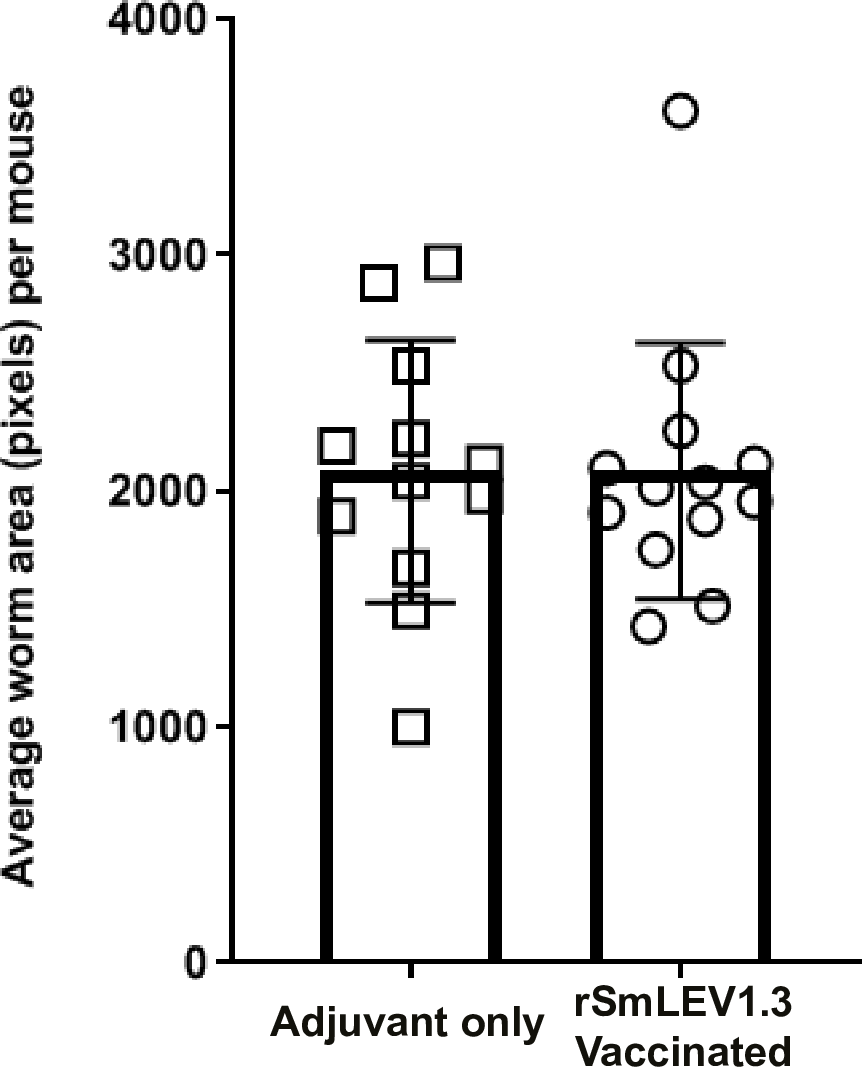

Supplement: S7 Fig — Upon termination of the vaccine regime (7 weeks post cercarial challenge), adult worms were perfused and anaesthetised for photographing. Images were processed for pixel counting in ImageJ [66]. No significant difference in worm size was seen between vaccinated (mean worm area per mouse: 1,934 pixels) and control animals (mean worm area per mouse: 2,081 pixels). The line and whiskers represent the mean with the standard deviation. Individual data points are also plotted. (TIF) [file pntd.0009981.s007.tif]

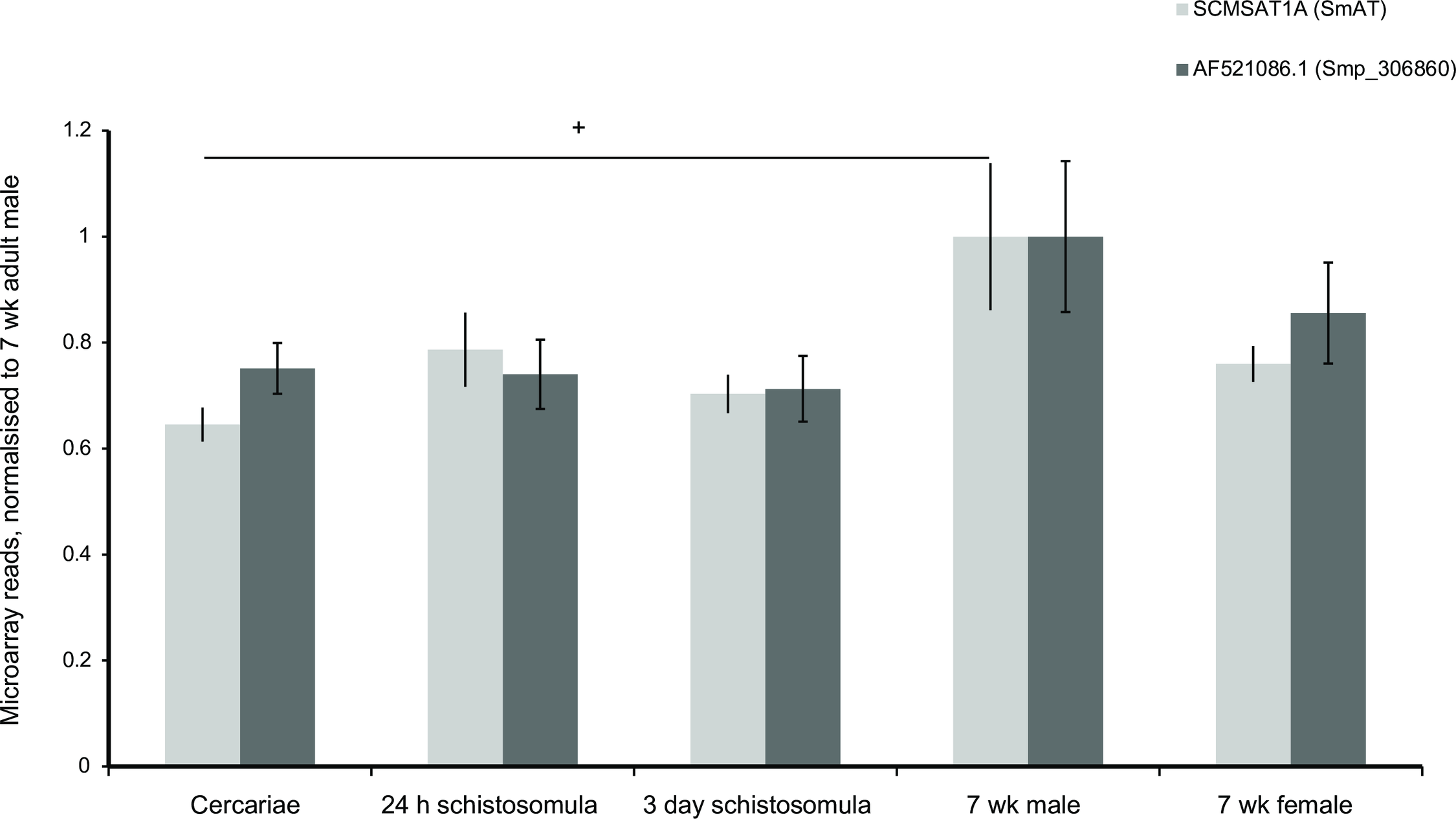

Supplement: S8 Fig — Data from the previous S. mansoni DNA microarray [19] was mined to identify a suitable reference gene across cercariae, 24 h schistosomula, 3-day schistosomula, 7-week male- and female adult worms. The contig AF521086.1, mapping to Smp_306860 exhibited the lowest variation in abundance, with no statistically significant differences between any of the lifecycle stages. SmAT (alpha tubulin) was significantly higher in 7-week adult male worms than in cercariae (p<0.05, +). The specific life-cycle stage is shown along the x-axis, mean normalised fluorescence units ± sem (standard error of the mean) were normalised to 7-week adult males (shown on the y axis) to enable the two contigs to be represented together. (TIF) [file pntd.0009981.s008.tif]
