# Supplementary material for: Schistosoma mansoni Larval Extracellular Vesicle protein 1 (SmLEV1) is an immunogenic antigen found in EVs released from pre-acetabular glands of invading cercariae
Source: PLoS Negl Trop Dis. 2021 Nov 18;15(11):e0009981. doi: 10.1371/journal.pntd.0009981 (PMC8639091; doi:10.1371/journal.pntd.0009981)
Supplement: Attachment — Submitted filename: Response to reviewers comments.docx [file pntd.0009981.s012.docx]

Dear PLoS NTDs,

We wish to thank the editors and reviewers for their work and response. Please find attached our response to each reviewers comments (labelled “A:”). As requested we have uploaded the new manuscript once with track changes and one without. No changes were made to figures, so these remain unaltered.

We also ask whether there is any way we may be able to pay the open access publication for this publication as soon as possible due to a grant ending on 15^th^ December.

Kind regards,

Dr Tom Gasan and Dr Iain Chalmers

Reviewer #1: This is a really nicely written characterization of an abundant S. mansoni EV protein. I commend the group for their thorough and straightforward analysis and presentation of a considerable amount of work. Some comments:

134: Can the transcript abundance analysis by itself tell us anything about which transcripts (of the various splice variants) are present and in what proportions?

A: Unfortunately not, the SmLEV1 DNA probe (contig_44) matches to a sequence spanning exons 10 and 11. This is one of the constant regions of the *smlev1* transcript, therefore the DNA Microarray data only tells us the total abundance of all *smlev1* isoforms. We made steps to address this in the QPCR experiment as described in the manuscript. As this examines the abundance of the variable exons and not each entire transcript we can only make inferences about the abundance of splice variants.

146: Report whether the pattern of expression of exon 1 (as measured by qRT-PCR amplification) matches that shown from RNA-seq read analysis, in figure 2A?

A: We thank the reviewer for this suggestion, it has been addressed in line 167 (new numbering). Indeed, the Q-RTPCR amplification of exon 1 closely resembled the cercarial dominant expression pattern observed in the RNA seq, and DNA microarray.

159: The abundance of exon 2,5,8 transcripts in females and males seems (roughly) the same (fig 2B), yet the smlev1 RNA-seq data shows substantially higher numbers for males (Fig 2A). Can the authors explain this discordance?

A: The RNA-seq data shows total abundance of all *smlev1* isoforms, with males expressing much more total *smlev1* than females. However, the abundance of each exon is measured by % of exon 1 (i.e. % of total transcripts) and thus appears much the same, with exon 5 showing significant differences between the sexes.

269: The total tally (n) in fig 5A is 42; where does the number 46 come from?

A: We apologise for this typo, 42 is correct. Changed in text

598: Report how the cercariae were fixed and permeabilized?

A: We thank the reviewer for this improvement to the methods section, this is now addressed in line 485 (new numbering).

Fig 6D: Any thoughts as to why the levels of IgE differ (significantly?) between the groups pre-vaccination?

A: We thank the reviewer for highlighting this difference. Unfortunately we have no clear explanation. The mice were purchased at the same time, from the same supplier, and housed in the same facility (i.e. the same room).

Fig 7D: Did the authors only measure the volume of 10 granulomas per group? If yes, doesn’t this undercut the veracity of their conclusion? Why so few?

A: We thank the reviewer for pointing out that the Fig 7 legend was unclear. We measured 10 granulomas per mouse. This is corrected in Fig 7D legend. “Each data point is the average granuloma volume per mouse, from 10 individual granulomas”

S1 Fig: Can the authors identify exon 5.2 in the S. mansoni genome?

A: We thank the reviewer for this insightful comment. This anomaly seems to only present in members of the *S. haematobium* clade. When searching for LEV1 homologues in schistosome species with a draft nature to the genome (*S. mattheei, S. curassoni)* it was necessary to BLAST each *S. mansoni LEV1* exon sequence in turn (rather than the entire CDS of SmLEV1). Exon 5 of SmLEV1 repeatedly mapped to two locations in all the genomes of the *S. haematobium* clade, with comparable e values and % ID. This was checked with iterative searches with the *S. haematobium* LEV1 sequence. We therefore deduced that there was an exon duplication in this clade, and represented both exon 5 and exon 5.2 in the correct sequential order.

S2 Fig: Why show us contig_44 here?

A: Contig_44 is represented in this figure, to highlight the location of the corresponding sequence on *smlev1*. This is of particular importance given that it maps to exon 10/11 – a region that is constant across all SmLEV1 isoforms.

S7 Fig: Consider reporting what area 2,000 pixels (say) actually represents.

A: Unfortunately we did not correlate pixels with e.g. cm^2^ in the original experiment, so the units must stay as pixels.

S8 Fig: Say what SmAT is.

A: “Alpha tubulin” has been added to the figure legend.

Reviewer #2: This is a well written and interesting manuscript that investigates for the first time an EV protein termed SmLEV1. The authors demonstrate that this is the most abundant protein in S. mansoni schistosomula EVs and that the protein is only found in the Schistosomatidae. The protein is found to be differentially spliced across the parasite life cycle and is localised to the pre-acetabular glands of cercariae. Interestingly Ugandan fisherman infected with the parasite display strong IgG1 responses to SmLEV1. The finding that vaccinated mice display reduced granuloma volumes is exciting and hints at potential therapeutic routes emerging from this work. The study seems to have been thoroughly conducted and the methods are well described. The Introduction provides an excellent grounding in work done to date, the results are well presented, and the discussion is appropriate and interesting. The data support the main conclusions of the paper. Overall, this paper will
be of interest to those in the schistosome research community and those studying aspects host-parasite interactions and EVs in general.

Minor points:
Author summary: I feel it should state somewhere in this section that schistosomes are human blood parasites.

A: Corrected. In line 71 we have stated “human skin”. In addition we have included the phrase “human infection” to line 80.
I presume that somules were not stained with anti SmLEV1 antibodies? Although not essential this may have revealed the extent to which the protein is released during transformation and somule development over 72 h.

A: We agree that this would have been interesting data. Unfortunately, we found somules much harder to fix and permeabilise using our protocol. Also when these IHC experiments were being conducted, the specific changes in expression of the first 72 hours was not yet clear to us, therefore we focused on cercariae.
Could a different adjuvant have induced a stronger immune response? This could be discussed briefly in relation to the different adjuvants used in murine-based vaccination trials.

A: We thank the reviewer for the comment regarding adjuvants. We regarded the immune response as acceptable as measured by the level of SmLEV1 Ab induced post-vaccination. Whilst indeed there are numerous adjuvants available, we chose to go for an adjuvant that has been used in previous *S. mansoni* vaccination studies (DOI: 10.1016/j.micinf.2012.10.007. DOI: 0.1038/srep15069. DOI: [10.1590/s0074-02762006000900060](https://doi.org/10.1590/s0074-02762006000900060)). If we are to investigate the anti-pathology potential of an SmLEV1 vaccine, we would more thoroughly compare other adjuvants.

There is some discussion (line 405 onwards) of the SMLEV1 protein potentially being present at the tegument of adult worms. Did the authors try to concentrate this worm fraction by stripping the tegument and running a greater amount of protein on a western blot? Immunolocalisation may also have helped here.

A: We didn’t try probing teguments extracts specifically, but we did greatly increase the primary Ab concentration (x1000) and amount of total protein loaded on a WB and were unable to detect SmLEV1 in adult worm extracts.

In addition, other soluble proteomic studies (mass spec) of total worm extracts are unable to identify SmLEV1 (references 9-11, 40, and 41). We have added an additional ref (reference 42) for the lack of detectable SmLEV1 in tegument protein extracts.

The discordance between the very specific transcript localisation and lack of protein in adult worms is interesting for further studies into SmLEV1

Minor, typographical etc:

A: We thank the reviewer for identifying these. These are now all corrected.

--------------------

Reviewer #1: Yes: Patrick Skelly

Reviewer #2: No
